# Microgravity Effects on the Matrisome

**DOI:** 10.3390/cells10092226

**Published:** 2021-08-27

**Authors:** Ludmila Buravkova, Irina Larina, Elena Andreeva, Anatoly Grigoriev

**Affiliations:** Institute of Biomedical Problems, Russian Academy of Sciences, Khoroshevskoye Shosse 76a, 123007 Moscow, Russia; irina.larina@gmail.com (I.L.); andreeva_er@mail.ru (E.A.); grigoriev@imbp.ru (A.G.)

**Keywords:** space flights, microgravity, matrisome, connective tissues, stromal lineage cells, transcriptomics, proteomics

## Abstract

Gravity is fundamental factor determining all processes of development and vital activity on Earth. During evolution, a complex mechanism of response to gravity alterations was formed in multicellular organisms. It includes the “gravisensors” in extracellular and intracellular spaces. Inside the cells, the cytoskeleton molecules are the principal gravity-sensitive structures, and outside the cells these are extracellular matrix (ECM) components. The cooperation between the intracellular and extracellular compartments is implemented through specialized protein structures, integrins. The gravity-sensitive complex is a kind of molecular hub that coordinates the functions of various tissues and organs in the gravitational environment. The functioning of this system is of particular importance under extremal conditions, such as spaceflight microgravity. This review covers the current understanding of ECM and associated molecules as the matrisome, the features of the above components in connective tissues, and the role of the latter in the cell and tissue responses to the gravity alterations. Special attention is paid to contemporary methodological approaches to the matrisome composition analysis under real space flights and ground-based simulation of its effects on Earth.

## 1. Introduction

In recent decades, significant progress has been made in the understanding of basic principles of the cellular response to the alterations of gravitational stimulus due to the development of cellular and molecular biology approaches [1,2,3,4].

According to the current mechanochemical hypothesis, integrins and other cell surface receptors play an important role in the physical interaction between extracellular matrix (ECM) and cytoskeleton. Mechanically forced deformations in these linked structures switch on/off various intracellular molecular events involving the cytoskeleton structures and associated signal transduction cascades [4,5,6,7].

ECM, as a counterpart of the gravisensivity network, remains much less studied than the cell surface structures, cytoskeleton and related intracellular events. At the same time, it is well known that the skeleton with abundant ECM as well as muscle tissues are most sensitive to space flight microgravity [2,8,9,10,11]. It is obvious that progress in the study of the response to microgravity and the development of approaches to the prevention of the negative effects of gravity deprivation is impossible without considering the role of ECM.

## 2. Current Concept of the Extracellular Matrix as a Complex of Structural and Regulatory Molecules: Matrisome

ECM is a complex three-dimensional macromolecular network that provides support for tissues and organs in multicellular organisms. According to the classical statements, all ECM components are composed of fibrillar (insoluble) and amorphous (soluble) ones [12]. Structured fibrillar part consists of collagens, elastins, fibronectins, and laminins, forming ECM fibers and fibrills. Amorphous (soluble) components primarily are represented by glycoproteins and proteoglycans. Besides, the ECM may be also distinguished as pericellular and interstitial compartments differing in their structure/composition [13]. The repertoire of ECM components is tissue specific.

The ECM functions are far beyond the physical support of the integrity and elasticity of tissues. It is a dynamic structure that is continuously being reconstructed to control homeostasis, regulating various cellular processes such as proliferation, migration, differentiation, viability, and morphogenesis [14,15,16]. Currently, ECM is considered as a multicomponent structure, including a complex of structural molecules, remodeling molecules, and ECM-associated biologically active metabolites.

**Figure 1:**
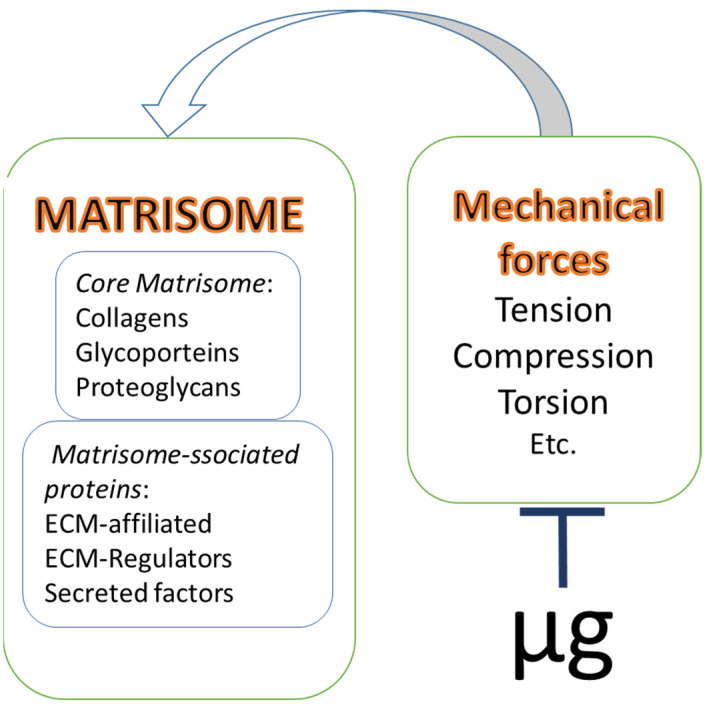
Matrisome and microgravity. According to the current concept an ensemble of ECM structural (core) elements and ECM-associated proteins represents a complex structure called matrisome. The activity of matrisome elements is governed by different mechanical forces, strongly affected by microgravity (µg).

In 1984, Martin et al. used the term “matrisome” to describe the structural organization of the components of basement membranes, defining it as “supramolecular complex forming the functional ECM units” [17]. Naba and colleagues have suggested to expand the concept of matrisome considering the latter as an ensemble of ECM structural (core) elements and ECM-associated proteins. According to the authors, the latter include linked soluble molecules (growth factors, cytokines, etc.), regulatory molecules (proteases and their inhibitors), and ECM-affiliated proteins (Figure 1) [18,19]. Since many ECM molecules are insoluble and difficult to isolate, various proteomic methods are a useful tool to predict the relevance and structure of proteins to certain matrisome compartments, based on the specific domains of matrisome molecules. The above has significantly expanded the number of ECM-associated components [18].

The data on most principal ECM and ECM-associated components as well as their involvement in the physiological processes have been revised in detail [14,20,21,22,23,24,25,26,27,28,29]. Below we will shortly summarize the available information focusing on matrisome-related molecules.

Main ECM structural components belonging to collagens’ superfamily should be mentioned first of all among the core matrisome molecules. This superfamily includes 28 molecules composed of 46 various polypeptide α-chains forming triple helices. Fibrillar collagens are abundant in the organs resistant to stretching (skin, bones, ligaments, and cartilage). In addition to the primary structural function, collagen networks transmit intercellular signals, which affect various cellular functions, including migration, adhesion, angiogenesis, and tissue development and repair [23]. Several proteoglycans, glycoproteins, and enzymes are involved in the formation of collagen networks surrounding the cells. Thus, decorin and biglycan are required for the proper fiber formation [30]. Lysyl oxidase (LOX) enzyme forms cross-links between collagen fibrils, giving mechanical strength to the structure [21]. 

The core matrisome includes proteoglycans—the most important structural and functional ECM components. Recently, it has been proposed to divide proteoglycans into four families: intracellular, cell-surface-bound, pericellular, and extracellular proteoglycans [27]. These high-molecular compounds consisting of covalently bound proteins (5–10%) and glycosaminoglycans (GAGs) (90–95%) represent the “ground substance” of the ECM. Proteoglycans are filling spaces between the cross-linked fibrillar ECM macromolecules thus mediating cell–matrix interactions. The GAGs are responsible for the hydration of the extracellular environment and support of tissue turgor and lubrication [24].

Glycoproteins comprise approximately 200 matrisome “core” molecules [19]. This is the largest group, whose components perform numerous functions, including ECM fibril assembly enhancement, involvement in adhesion, and growth factor binding [26]. Among glycoproteins, fibronectins, laminins, elastins, fibulins, thrombospondins, tenascins, are the most well studied ones. Due to multi-domain structure, stable soluble fibronectin fibrils simultaneously bind cellular receptors, collagens, proteoglycans, and other focal adhesion molecules mediating the assembly of other ECM protein components [20,29]. Laminins in combination with type IV and V collagens, play an important role in the structural organization of the basement membrane [14]. In addition, laminins interact with the cell surface through binding to integrins, dystroglycans, and sulfated glycolipids, which contributes to the modulation of adhesion, differentiation, migration, phenotype stability, and resistance to apoptosis [26]. Elastin as insoluble polymer of tropoelastin monomers determines the elastic properties of tissues and is abundant in the skin, lungs, ligaments, tendons, and blood vessels [22]. Glycoproteins of tenascin family are known to be strongly involved in modulation of cell adhesion, migration, and growth [28]. Thrombospondins bind to ECM components, such as heparan sulfate, and cell membrane receptors, thus providing cell–ECM interaction [25]. Moreover, thrombospondins promote collagen fiber formation [31]. 

Matrisome-associated molecules include heterogeneous groups of proteins. Among them, there are ECM-regulators which are of significant importance for ECM remodeling. ECM-regulators are presented by ECM-remodeling proteases, and their inhibitors. These enzymes exhibit various activities including ECM-crosslinking (lysyl oxidases, transglutaminases), ECM-modification (sulfatases, extracellular kinases), and ECM-degradation (matrix metalloproteinases (MMPs), serine proteases (plasminogen/plasmin), cysteine proteases (catepsins) [18]. The ECM dynamics are orchestrated by cells via the synthesis of ECM enzymes and relevant inhibitors in response to various signals [32]. Proteolytic enzymes are able directly degrade matrix proteins into small peptides with signaling functions. To maintain tissue homeostasis under normal development, the activity of proteases and its inhibitors must be controlled and balanced. Disorders of this protease–anti-protease balance can cause various pathological processes, including tumor progression [33]. 

Besides the direct effects, ECM molecules also regulate cell activity indirectly, due to the capacity to deposit and release growth factors. Some matrix components have been shown to be able to bind these mediators, thus making them insoluble and inactive. Glycoproteins, collagens, and proteoglycans can bind growth factors such as TGF-β and insulin-like growth factor (IGF), fibroblast-, hepatocyte-, and endothelial growth factors (FGF, HGF, and VEGF, respectively) [34]. As an example, fibronectin links specifically to a variety of growth factors (VEGF, HGF, PDGF, etc.) [35,36]. TGF is demonstrated to bind specifically to fibrillins and to fibronectin-rich matrices [37,38]. Due to matrix-remodeling enzymes, these factors can be released, forming local biochemical gradients that control pattern formation during developmental processes [32,39]. According to Hynes and Naba [18], so called ECM-affiliated proteins may be also considered as ECM-associated. These molecules could be presented in soluble as well as in ECM-associated forms (semaphorins, plexins, collagen-related molecules and homologs) or to be transient components of ECM as annexins or galectins [40]. 

One of the most important properties of ECM that determines its direct effect on cell functions is executed through the formation of stable links with specialized cell surface receptors. These primarily include integrins, a large family of heterodimeric transmembrane receptors that interact with ECM components. Combinations of 18 α-subunits and 8 β-subunits yield up to 24 different heterodimers expressed in different cell types with the overlapping substrate specificity. Most integrins bind to RGD-containing ECM proteins (such as fibronectin, fibrinogen, and vitronectin) [5,41,42]. 

The composition and biomechanical properties of ECM are highly specialized according to the requirements of the structure and functions of certain tissues and organs. Nevertheless, despite the significant organotypic diversity of ECM, the above compartments of structural core and matrisome-associated proteins are always present in the matrisome. Therefore, the proposed classification is a convenient tool that allows comprehensive assessment of ECM state under physiological or pathological conditions in various tissues.

## 3. Matrisome of Connective Tissues

Loose and dense connective tissues are the most significant ECM depot in the body. In this regard, it is interesting to briefly discuss the features of the matrisome of these tissues, and the matrisome-associated activity of the stromal lineage cells, i.e., multipotent mesenchymal cells (MSCs), and their more committed progeny, such as osteogenic and chondrogenic lineage cells. 

Matrisome characterization in vivo is quite challenging due to the peculiarities of the physico-chemical properties of ECM molecules and the difficulties of its isolation. ECM proteins have a large molecular weight, are poorly soluble, and usually have a number of post-translational modifications [43,44]. Moreover, the presence of a large number of readily soluble cytoplasmic and mitochondrial proteins can interfere with the determination of ECM proteins. Therefore, proteomic methods, along with bioinformatic analysis, are very requested in the analysis of ECM. 

Low-committed stromal progenitors can be isolated from almost any tissue with a well-developed microvasculature, where they are found in the periendothelial spaces. Using proteomic analysis, 150 ECM proteins, including 91 core matrisome proteins and 59 matrisome-associated proteins, were annotated in the soft medulla microvasculature and brain microvessels [45]. This is consistent with the existing in silico assumption of matrisome being represented by 100–150 proteins in any tissue [43]. Meanwhile, only 24 proteins were similar in these two loci. In the microvasculature, the main ECM producers are pericytes, cells identical or cognate to MSCs, therefore the above data can be considered as an MSC matrisome characterization. 

A detailed study of the native dog tendon and ligament matrisomes, as well as of 3D-cell constructs derived from these tissues, were performed by Kharaz et al. [46]. The ligament matrisome proteins were annotated as collagens (11), glycoproteins (27), proteoglycans (13), ECM regulators (10), ECM-affiliated (11), and ECM-secreted factors (9). Collagens (11), proteoglycans (13), glycoproteins (30), ECM regulators (11), and ECM-secreted factors (8) were found in the tendons. The profiles of matrisome proteins were even more similar in in vitro 3D constructs obtained from cells isolated from ligaments and tendons, respectively: collagens, 10/10; glycoproteins, 18/18; ECM regulators, 11/11; proteoglycans, 8/9; ECM-affiliated, 11/13; ECM-secreted, 5/6. The qualitative signature of the proteins varied significantly. The ligaments were enriched in collagen type II-alpha 1, aggrecan, chondroadherin, while thrombospondin 4, collagen types III and XII, asporin; aggrecan, and versican were predominant in the tendons. Tendon and ligament-derived 3D constructs contained similar principal core-matrisome and matrisome-associated proteins as native tissues, including collagen I, III, V, VI, and XII, XIV, decorin, biglican, asporin, osteoglycin, fibronectin 1, and fibrillin. These findings indicate that 3D constructs are able to reproduce specific tendon and ligament ECM proteins. However, the pronounced qualitative differences as between the matrisomes of the native tissues have not been shown for 3D constructs [46].

The proteomic analysis of the muscle and skeletal morphogenesis of a mouse embryo forelimb detected 122 matrisomal proteins, with their ratio varying significantly depending on the embryogenesis stage. Most of the proteins were represented by collagens and glycoproteins [47].

Li et al. demonstrated that osteodifferentiation of osteoblasts was stimulated in the co-culture with fibroblasts. It was accompanied by formation of a specific ECM profile, in which 178 proteins were identified, 80 of them belonging to the core matrisome and 98 to matrix-associated proteins [48].

According to in silico data, the matrisome quantitative composition is quite conservative [19,40]. The studies cited above have demonstrated the consistency of connective tissue matrisome quantitative parameters and the significant variability of its qualitative characteristics in vitro and in vivo. From the point of mechanical stimulus reception, connective tissue matrisome, including muscles, skeleton, and soft connective, is the most important and, probably, the primary effector that triggers the gravity-dependent response.

The suggestion that bone tissue formation and remodeling are regulated by mechanical forces was generated over 100 years ago and is acknowledged as Wolff’s law [49]. It was postulated that the orientation of bone trabeculae correlated with the direction of load caused by daily physical activity. Based on the above, bone tissue was suggested to be able to adapt its architecture to the mechanical environment [50]. Later, the numerous studies demonstrated the effects of various mechanical stimuli, such as tension/compression, twisting, shear stress, and gravitational load/unload, on the physiology and pathology of individual cells, tissues, and organs of musculo-skeletal system (Figure 1) [51,52,53]. According to Ingber’s tensegrity model, the continuous series of molecular struts (microtubules, cross-linked microfilament bundles), cables (contractile microfilaments), and ropes (intermediate filaments) in the form of a discrete cytoskeleton, is a principal intracellular effector of above-mentioned impacts [6]. The rearrangement of cytoskeleton structures governs the cellular response to those extremal influences. ECM components could act as a kind of “primary mechanical sensor” in the extracellular space. ECM stiffness, elasticity, and viscosity (hydration) determine the efficacy of mechanical signal transduction to cell receptors, including integrins, which results in the cytoskeleton rearrangement and changes in gene expression [42]. Therefore, ECM components are involved in the regulation of cell proliferation, migration, differentiation, and apoptosis [54].

## 4. Connective Tissue Matrisome and Microgravity

### 4.1. Space Flight Experiments

It is well established that a significant decrease in bone mineral density resulting in osteopenia is one of the most pronounced effects of gravity deprivation in humans and animals [8,9]. The severity of the observed bone microstructure rearrangement was found to largely depend on the bone location relative to the gravity vector [8,55]. The local bone mass loss under mechanical stress deficiency or microgravity suggests that the mechanical signal reception (or its absence) can also be performed at the cellular level. Other organs with a well-developed connective tissue component, such as muscles, blood vessels, and skin [56], can also undergo negative changes under the influence of microgravity, including atrophy [57].

The mechanisms of gravisensitivity of stromal lineage cells of different commitment in the above connective tissue compartments are being intensively studied. At a moment, the attention is mainly focused on the molecular cascades associated with the mechanotransduction from the extracellular space to the cytoskeleton [4,5,6,7]. The involvement of matrisome as a gravireceptor has not yet been adequately studied. Meanwhile, the already existing data elicit some changes in the main matrisome compartments.

The opportunities to execute the experiments on board of unmanned or manned space vehicles are extremely limited. Therefore, a few ones have obtained data on the changes of the matrisome elements during space flights. On ISS, a single study on the whole organism - a medaka fish, was performed [58]. At flight day 1 live-imaging detected an excessive fluorescence of a core glycoprotein DsRed-osteocalcin in pharyngeal bone osteoblasts versus ground-based control. At flight days 5 and 8, the increase fluorescent signal was sustained. High throughput sequencing analysis of pharyngeal bones of juvenile fish at day 2 after launch detected upregulation of two osteoblast-related genes *COL10A1* and osteocalcin (*OCN*) as well as ECM-remodeling *MMP9* [58].

In several space missions, in vitro experiments of different duration were performed using the osteogenic precursors (Table 1). Both in Foton 10 satellite unmanned [59] and STS-54 [60], STS-59 [61], STS-65 [62] manned missions a downregulation of *OCN* starting from flight day 5 till day 12 was detected. In addition, experiments at Foton 10 and STS-59 demonstrated a downregulation of the master core protein, *COL1A*. In a recent experiment at SJ-10, downregulation of several genes encoded core matrisome and upregulation of *MMP1* was observed after 2 days of flight. The inhibiting effect on *COL1A2* was detected in the same mission after 5 days [63]. Based on inflight observations, it can be assumed that microgravity negatively affects core matrisome proteins at the transcriptional level. The increased DsRed-osteocalcin protein fluorescent signal described in medaka fish may indicate the post-translational effects of microgravity.

### 4.2. Ground-Based Simulations

Various approaches have been developed to simulate spaceflight effects under ground-based conditions, including experiments at the physiological level or individual cells (Table 1).

In animal experiments, anti-orthostatic suspension of rodents (mice or rats) is the mostly demanded “unloading” model. In several studies, low-committed stromal precursors (MSCs) were isolated from the hind limb bone marrow from suspended and control animals. The subsequent cultivation demonstrated a decreased osteogenic potential of MSCs associated with a downregulation of osteoblastic commitment-related genes [64], including core glycoprotein ostepontin (*OPN*), and a decreased mineralization of the ECM [65]. It is important to note that after a short-term suspension (5 days), a decreased *OCN* transcription was detected in cultured MSCs [60], which was consistent with the effects described in osteo-precursor cultures in spaceflights. The same authors found an increased *OCN* expression in MSCs isolated and expanded after 14 days of suspension [60]. Since the multidirectional changes in transcription of *OCN* were described in the same experiment, this may be an indication of time-dependent response of ECM-associated genes.

To study the effects of microgravity on the cells, various devices have been developed to simulate the absence of gravity. These include devices that provide fast and slow rotations of biological objects (2D and 3D clinorotation), three-dimensional dynamic rotation (Random Positioning Machine (RPM), and large-volume rotating-wall vessels (RWV) [66]. All ground-based simulations provide a randomization of position of cells relative to the gravity vector. The limitations in each case do exist. 1D/2D clinostat/RWV cancel the directionality of the gravity vector attenuating but not eliminating the gravity. The shift in weight distribution can cause mechanical and bending stress. Using RPM, high quality microgravity conditions down to 10^−^^4^ g can be obtained. It strongly depends on the combination of rotation speed and the distance from the center, thus, the experimental conditions must be carefully set [67].

Such devices provide the opportunity to identify the mechanisms of influence of the altered gravitational environment on cells as well as to adapt methodological approaches before using them in the spaceflights.

RWV experiments of various durations showed no change or a decrease in the transcription of the main core collagen I and a number of core glycoproteins (Table 1). These effects were similar in the low committed [68,69] or osteo/chondro-induced stromal progenitors [70,71,72], osteoblasts [73], and osteocytes [69].

Using gravity vector randomization approaches with 2D/3D clinostats or RPM devices, multidirectional changes in the transcription of core protein genes and ECM-associated and affiliated molecules were found (Table 1).

An upregulation of genes encoding core matrisome proteins was demonstrated in a number of papers [3,74,75,76,77,78,79,80,81,82]. In juvenile human fibroblasts, 3 days SMG (RPM) induced an increase of transcription of core basement membrane collagen IV, and transcription/translation of the principal core glycoprotein fibronectin, as well as of MMPs involved in ECM remodeling [3]. Upregulation of several genes encoding core proteins and glycoproteins in human adipose MSCs was described after 4 days of RPM exposure [74]. Though RPM exposure till day 10 affected fewer differentially expressed matrisome genes, a part of core matrisome encoded genes were still significantly upregulated, while genes encoding ECM-degrading enzyme and its inhibitors were downregulated [75]. 

Besides, it was demonstrated a time-dependent dynamic of the transcriptional activity of matrisome genes under simulated microgravity exposure [76,77]. In comparison to static conditions, core collagens were downregulated in bone marrow MSCs after 5 days at RPM, there were no differences after 10 days, and these genes were significantly upregulated after 20 days. At the same time, after 10 and 20 days of exposure, the expression of core glycoprotein osteomodulin (*OMD*) that regulates osteoblast adhesion was reduced as well as ECM-associated growth factors, while *OCN* transcription did not change [77]. Regardless of exposure time, a decreased transcription of *OMD* was demonstrated in human murine and preosteoblasts [77,78].

Long-term microgravity simulation with RPM was demonstrated to have different effects on the efficacy of mineralization of the ECM by stromal cells of different commitment levels. In osteocommitted MSCs, the deposition of calcium was reduced, and in osteoblasts, on the contrary, it was increased [77]. 

In MC3T3-E2 lineage osteoblasts, 3 days of 3D-clinorotation was accompanied by upregulation of genes encoding enzymes that provide extracellular posttranslational modification of collagen fibers, and the increase of functional activity of the above enzymes [79]. An increased expression of core collagen gene was detected after 7 days of human adipose MSC 2D clinorotation [80]. 

On the other hand, the data are available on the suppressive effects of simulated microgravity on matrisome compartments. The downregulation of glycoprotein fibrillin (*FBN1*) and *MMP1* was noted after 7 days 2D clinorotation of human adipose MSCs [80] as well as of core *COL1* and *FBN1* in MC3T3-E2 murine osteoblasts following 7 days of 3D-clinorotation [81]. In addition to changes in core proteins, a decreased transcriptional activity of genes encoding molecules associated with ECM metabolism like transcription factors *cbfa1/RUNX2* was noted [82].

Thus, the available in vivo and ex vivo information on the effects of real and simulated microgravity on the ECM and related molecules indicate the direct involvement of the matrisome components in the adaptation of stromal lineage cells to gravity deprivation. The short-term exposures provoke multidirectional alterations in ECM-related gene activity, which ensures the adaptation. In general, the direction of changes of the matrisome gene transcription does not change critically depending on the commitment level of stromal progenitors. However, the above data by Gershovich et al. have shown that the differences in the ECM response can be reflected at the ECM maturation level [77]. In this regard, the search for integral markers that make it possible to assess the changes in the body matrisome under various extremal conditions is becoming more and more demanded. The application of rapidly developing omics is one of the most promising approaches in the above direction.

## 5. Proteomic Profile of Human Matrisome-Associated Proteins under Real and Simulated Microgravity

Proteomic approach attracts considerable attention in the study of physiological and pathological changes. Several recent reviews describe the effect of microgravity on the animal and human proteomes [83,84,85,86,87]. Over the past few years, mass spectrometry has become the method of choice for the characterization of ECM composition [40,88,89] and has been shown to offer new bioinformatic approaches of translating data from the putative biomarkers to the elucidation of new therapeutic targets [90,91,92]. The experimental strategies, new bioinformatic tools, and methods for matrix isolation have been described for the research on the ECM composition and mechanisms of degradation/renewal [40,43,44,93,94]. The new MatrisomeDB version contains selected proteomic data from 17 studies with ECM from 15 various tissues and includes 847 human ECM proteoforms and over 350,000 peptide-to-spectrum matches [44].

The proteomic data on matrisome components are considered to be of diagnostic and prognostic values in clinical studies. A comparison of core ECM and ECM-associated molecules’ data from the human carotid endarterectomy samples demonstrated the differences in the proteome and gene expression in symptomatic and asymptomatic atherosclerotic patients, including MMP-9, chitinase 3-like-1, calcium binding protein S100 A8 (S100A8), S100A9, cathepsin B, fibronectin, and galectin-3-binding protein [95]. Proteomic analysis revealed the loss of aggrecan and several small leucine-rich proteoglycans, with a compensatory increase in collagen I during ECM remodeling in varicose veins, though there were no significant alterations of gene expression. These data suppose that the remodeling process associated with venous hypertension mainly occurs at the translation level, rather than at the transcription one. [90].

Investigation of the effects of spaceflights and ground-based simulations on the proteomics of matrisome in healthy subjects is of great interest. Several experimental modes that involve healthy volunteers are used to simulate the effects of certain spaceflight factors. These include various types of immersion and head-down tilt bed rest (HDT BR).

After 21 days of “dry” immersion, the altered levels of a number of proteins were detected in plasma with chromatography–mass spectrometric analysis. The identification of overrepresented processes, as well as processes and biological pathways was performed using the GO databases (biological processes, pathways, and KEGG). The significantly changed proteins were annotated as involved in ECM remodeling (alpha, beta, fibrinogen gamma chains), fibronectin, transtyretin), vitronectin and the cell morphogenesis regulation (alipoprotein A-I, prothrombin, alpha, beta, gamma chains of fibrinogen, fibronectin [96,97]. Hypokinesia is accompanied “dry’ immersion as well, and probably causes a protease/counter protease imbalance, which may be responsible for the ECM remodeling activation. Besides, after 21 day “dry” immersion a decrease in ECM proteoglycans, lumican and COMP, was also detected. COMP, as a cartilage structural protein, plays an important role in the ECM stabilization due to the interactions with collagen fibrils and other matrix components [98].

The blood COMP level is sensitive to physiological stress. The study of 14 day HDT BR demonstrated that the joint cartilage thickness was reduced during the experiment, followed by a decrease in the COMP level [99]. Liu et al. [100] reported the chondrocytes’ ability to respond to stress in the extracellular environment (possibly both mechanical and shear stress), which resulted in an altered expression of matrix proteins. Therefore, a decrease in the blood COMP level during “dry” immersion may reflect a reduced metabolic activity of cartilage matrix proteins in response to the lack of mechanical stimuli.

Based on the proteomic data of blood and urine samples collected in HDT BR and “dry” immersion of similar durations (21 days), GO pathways analysis was performed for proteins with significantly changed concentrations. It was demonstrated that ECM remodeling was the most significant process among them (in particular, collagen degradation) [101]. At day 21 of HDT BR, a significant decrease was observed in the levels of collagen I and XV alpha-chains, and cathepsin D that are associated with degradation processes and ECM collagen fibril assembly.

According to the “dry” immersion and HDT BR proteomic data comparison, at day 21, proteins involved in the ECM organization and metabolism were detected: endorepellin, nidogen-1, tenascin X, and vitronectin. Bioinformatic resources confirm that proteins endorepellin and nidogen-1 are primarily involved in the ECM degradation. The analysis demonstrated that the proteins that changed their blood levels under HDT BR have the catalytic activity functions (transferase and hydrolase activities). These findings provide information about the ECM structures’ involvement in the response to a reduced support load.

Urine, blood, and exhaled air condensate are the minimally invasive biological material samples available for proteomic research in astronauts. We believe that blood samples are the most preferable ones for the study of ECM components among the above liquids. da Silveira et al. have applied a multidisciplinary systemic biology analytical approach to determine transcriptomic, proteomic, metabolomic, and epigenetic responses to spaceflight [102]. Multi-omics datasets obtained from the analysis of an astronaut’s biological samples after a one-year space mission showed a significant enrichment of biological processes closely related to the functions of ECM.

Blood samples obtained from the Russian cosmonauts were examined by various proteomic mass spectrometry-based methods. Changes in the serum protein composition, including full-size proteins and the isoforms, fragments, metabolites, and peptides, after long-term spaceflights were characterized by direct mass spectrometry profiling after serum pre-fractioning using MB WCX magnetic particles. After a spaceflight, the peak areas of “acute phase” proteins, lipid metabolism, proteolytic enzymes and their inhibitors were shown to alter [103].

With semi-quantitative label free panoramic method, it was demonstrated that among 419 various proteins in cosmonauts’ blood plasma 17 proteins were significantly increased, while two significantly decreased after a prolonged space flight in comparison with pre-flight levels. In most cases, these proteins do not return to pre-flight baselines by day 7 after spaceflight. They are involved in the blood clotting system, ECM remodeling, and immune processes [104].

The quantitative changes in the cosmonauts’ blood proteome found using targeted MRM method with a panel of stable [^13^C]/[^15^N] isotope-labeled proteotypic peptides indicated that almost all proteins with the concentrations reacting to space flight can be combined into a network of interactions between the processes of regulation of protease activity, innate immunity, lipid metabolism, coagulation cascades, and ECM metabolism [86]. Latridis et al. suggest that these reactions may be triggered by extracellular signaling pathways of mechanotransduction [105]. Part of the group of functionally ECM-associated proteins detected in the samples at day 1 after landing in a reduced concentration, were found to return to baseline after 7 days, but another part of proteins retained significantly reduced concentrations [103,104]. The ANDCell program made it possible to identify biological processes involving ECM proteins that are modified by spaceflight factors (Table 2).

As evidenced from Table 2, the reorganization of the matrix structure due to MMPs, the ERK pathway regulation, cell–matrix adhesion, secretion, and assembly of ECM structures are the principal biological processes affected by spaceflight.

Proteomic analysis of urine samples from Russian cosmonauts after six-month missions detected 20 of the 256 proteins with altered levels [86,106]. However, the concentrations of most of them were returned to pre-flight levels within 7 days of the post-flight rehabilitation. At day 1 upon landing, seven proteins involved in ECM remodeling were identified among the significantly changing proteins. According to their functions in the physiological processes, they belong to the proteins involved in the musculoskeletal system metabolism. Thus, the level of osteodifferentiation and bone mineralization associated osteopontin was increased at day 1 upon landing and did not return to baseline after a further 7 days. Interestingly, several proteins not detected in urine samples prior to flight were increased acutely in post-flight: alpha-1-antichymotrypsin, N-acetyl glycosamine-6-sulfatase, cystatin-M, collagen alpha-1(I) chain, and vitronectin, granulin, and LDH beta chain. The above proteins are known to belong to the group of ECM-associated biological processes [106].

Therefore, the secretion and assembly of the matrisome components, its remodeling activity, as well as the ECM/cells associations are affected by spaceflight, primarily by the reduction of mechanical stress under microgravity.

## 6. Concluding Remarks and Further Directions

Gravity is the physical constant permanently affecting living organisms on Earth. Therefore, the gravity deprivation results in significant rearrangements at the macroscale (e.g., in organs and tissues), microscale (e.g., in single cell), and nanoscale levels (e.g., in molecular complexes or single proteins) [107]. The mechanisms of gravisensitivity have been actively investigated since humanity started space exploration. To date, due to research during space missions and ground-based experiments, the mechanism of mechanotransduction executing an urgent cell response to microgravity by transmitting a signal from the extracellular space to the cytoskeleton via integrins and further to the nucleus, has been studied essentially. Meanwhile, a number of questions are far from resolution. Namely, impaired mechanotransduction in entire cell population did not explain the appearance of two different phenotypes—clustered floating and adhered among the initially homogeneous substrate-dependent cells as described by Po et al. for MCF7 cells [108]. The authors suggested that the cellular response to gravity deprivation had occurred mainly at the expense of reversible cytoskeleton alterations rather than differential gene expression. Besides, it was demonstrated that after RPM exposure both non-tumorogenic mammary MCF-10A and poorly invasive breast cancer MCF-7 cells gave rise to two phenotypically different subpopulations [109]. However, in the case of normal MCF-10A there were two adhered populations, while cancerous MCF-7 popupation was composed of floating clusters and adhered cells. The authors succeeded to find out that cell detachment was critical for induction of apoptosis in cells under microgravity simulation, further accompanied by cytoskeleton and shape remodeling. Depending on the efficacy of adhesion, distinct Akt- and ERK-dependent pathways were upregulated in MCF-7 and MCF-10A cells, respectively [109]. No doubt, the interaction between cytoskeletal proteins and ECM is affected under gravity deprivation as well.

At present, the effect of gravity elimination on the components of the matrisome, a complex of structural and regulatory molecules, has been studied to a much lesser extent. As evidenced from the papers revised here, most data on the matrisome alterations of stromal lineage cells under the real and simulated microgravity are related to the transcriptional/translational activity of cells. The above data do not allow fully conceive the changes of the matrisome. Only few observations describing changes in ECM structures under gravity deprivation are available [75,80,110,111,112]. The challenges of matrisome isolation is an essential pitfall. Core matrisome structures, such as collagens, elastin, and laminins, are known to be insoluble, which complicates their isolation and analysis. In vivo methods for analysis of matrisome structures and its functions are still in their infancy. Wherein, the loss of bone and muscle mass is one of the most noticeable negative effects of spaceflights and ultimately, is associated with changes in the matrisome quality and quantity. Noteworthy, at the current level of technologies, there are limited approaches to regulate gravity magnitude either on Earth or during spaceflights. In this connection, different simulation approaches to examine matrisome state and its effects on the tissue and physiology levels are on demand in order to improve countermeasures of long-term gravity deprivation negative consequences.

Therefore, the need to expand the research of the matrisome as a gravity-responsive structure is becoming more and more urgent. In this connection, two directions are considered the most important ones.

First, it is the analysis of microgravity effects on the pre-existing matrisome. The goals of this milestone will include description of changes of existing ECM structures under microgravity; detection of biomechanical alterations and its effects on regulation of cell functions; elucidation fibrillar or amorphous core matrisome compartments are more sensitive; evaluation of activity of matrisome-remodeling enzymes; the characterization of depot function of ECM reflected in turnover of growth factors and water under microgravity; finally, how all the above matrisome changes affect cellular mechanotransduction.

Second, the inevitable microgravity-induced changes in the functional activity of stromal lineage cells associated with the production/degradation of matrisome components, as well as the maturation of de novo ECM, need to be investigated as well. In connection with cited above data on formation of distinct cell phenotypes under microgravity exposure, the similar potential among stromal lineage cells will be of particular interest in respect of differential matrisome response in those distinct subpopulations.

The combination of classical molecular and cellular biology methods with contemporary high-tech platforms, such as next generation sequencing (NGS) and OMICs opens up new prospects for elucidation of gravisensivity of matrisome both during spaceflights and ground-based simulations.

Getting the answers to these questions will make it possible to significantly advance in understanding the mechanisms of gravity-dependent responses in the cells and tissues, as well as to improve the countermeasures for preventing the negative effects of microgravity, especially related to the planned long duration spaceflights for the exploration of the Moon and Mars.

## Figures and Tables

**Table 1:** The effects of real microgravity and its simulation on matrisome components of stromal lineage cells.

Object	Cell Type	Duration	Matrisome			Exp. Approach	Study
			Core Matrisome		ECM-Associated Molecules		
			Collagens	Proteoglycans, Glycoproteins			
Space flight							
Medaka fish	OB, Ocl (ISS)	1, 5, 8 d	U: *COL10A1*	U: *OCN*	U: *MMP9*	DsRed live-imaging, GGA	[58]
Mouse	MG-63, osteoinduced (Foton 10)	9 d	D: *COL1A;* ND: collagen type I	D: *OCN*		RT-PCR, WB	[59]
Mouse	2T3 OB (STS-54)	6 d		D: *OCN*		RT-PCR	[60]
Chicken	OB (STS-59)	12 d	D: *COL1A;* ND: collagen type I	D: *OCN*		RT-PCR, WB	[61]
Rat	OB (STS-65)	5 d		D: *OCN*		RT-PCR	[62]
Human	OB, MSC-derived (SJ-10)	2 d	D: *COL1A1, -1A2, -3A1, -4A1, -5A1, -6A1, -8A1*	D*: TNC, COMP*	U*: MMP1*	RT-PCR	[63]
Human	OB, MSC-derived (SJ-10)	5 d	D: *COL1A1*			RT-PCR	[63]
Hind-limb suspension (HS)							
Rat	MSC-BM, tibia	5 d		D: *OCN*		RT-PCR	[60]
Rat	MSC-BM, tibia	14 d		U: *OCN*		RT-PCR	[60]
Rat	MSC-BM, femur	28 d		D: *RUNX2, COLI, ALP, OCN*	D: osteogenic potential; expression of osteoblast gene marker mRNAs under osteogenic conditions.	RT-PCR, HS	[64]
Mouse	MSC-BM, femur	7 d			D: mineralization	HS	[65]
	Rotating-Wall Vessel (RWV)						
Human	MSC-BM	7 d	D*: COL2A1, COL10A1*	D*: ON*		RT-PCR	[68]
Mouse	OB MC3T3-E1	1–14 d	ND: *COL1A2*	ND: *OCN*		RT-PCR	[68]
Mouse	OB/OC MLO-A5	1–14 d	ND: *COL1A3*	ND: *OCN, OPN*		RT-PCR	[69]
Mouse	MSC C3H10T(1/2)	1–14 d	ND*: OCN, OPN, COL1A4*	ND: *OCN, OPN*		RT-PCR	[69]
Mouse	OB MC3T3-E1	1 d		D*: OCN*		RT-PCR	[70]
Human	MG-63, osteoinduced	3 d	D: *COL1*	D*: OCN*		RT-PCR	[71]
Human	ChB, MSC-derived	21 d	D: *COL1*	D: *AGN*		RT-PCR	[72]
Mouse	OB	1d	ND: *COL1A2*	ND: *OCN, OPN*		RT-PCR	[73]
	Random Positioning Machine (RPM)						
Human	FB	3 d	U: *COL4A5*	U*: FN/*FN	U*: TGF*	RT-PCR, WB	[3]
Human	MSC-AT	4 d	U: *COL12A1, COL15A1, COL16A1, COL1A1, COL5A1, COL8A1*	U: *THBS1, THBS2, THBS3, LAMA, SPARC, TNC, VCAN, VTN;* D*: CLEC3B*		RT-PCR	[74]
Human	MSC-AT	10 d	D: *COL11A1;* D*:* collagenous proteins	D*: LAMB3, TNC;* U*:* non-collagenous proteins		RT-PCR, HC	[75]
Human	MSC-BM, osteoinduced	20 d	U: *COL1A1*	D: *OMD;* ND*: OCN*	D: ECM mineralization	RT-PCR, HC	[76]
Human	MSC-BM	5 d	D: *COL9A1, COL2A1*			RT-PCR	[77]
Human	MSC-BM, osteoinduced	10 d	ND*: COL1A1*	D*:* *OMD;* ND*: OCN*		RT-PCR, ICC	77]
Human	MSC-BM	20 d	ND: *COL1A1*; ND: collagen tot			RT-PCR, ICC	[77]
Human	OB	20 d			U: ECM mineralization	HC	[77]
Mouse	2T3 OB	3 d		D: *OMD*		RT-PCR	[78]
	2D, 3D-clinorotation						
Mouse	MC3T3-E2	3 d			U: *PLOD1, PLOD2;* U: enzymes activity	RT-PCR, enzyme assay	[79]
Human	MSC-AT	7 d	U*: COL1 COL3*	D: *FBN1*	D: *MMP1*	RT-PCR	[80]
Mouse	MC3T3-E1	7 d	D: *COL1A1*			RT-PCR	[81]
Rat	MSC	1–4 d			D: *cbfa1/RUNX2*	RT-PCR	[82]

Abbreviations: cell types: MSC—mesenchymal stromal cell; OB—osteoblast; OC—osteocyte; ChB—chondroblast; OCl—osteoclast. MSC sources: BM—bone marrow; AT—adipose tissue. Experimental approaches: RT-PCR; ICC—immunocytochemistry; HC—histochemistry; WB—Western blot; GGA—Hiseq global gene analysis. Matrisome and matrisome-associated molecules: COL—collagen, FN—fibronectin, FBN1—fibrillin; LAMA—laminin; OMD—osteomodulin; cbfa1/RUNX2—master transcription factor of osteogenic differentiation; OCN (BGLAP)—osteocalcin; ON (SPARC)—osteonectin; OPN (SPP1)—osteopontin; AGN—aggrecan; THBS—trombospondin; TNC—tenascin, VCAN—versican; VTN—vitronection; TNC—tenascin. The direction of the effects: D—downregulation; U—upregulation; ND—no difference.

**Table 2:** Spaceflight factors-affected biological processes involving matrisome proteins.

Protein	Uniprot Index	Biological Process (ANDCell)
Alpha-2-HS-glycoprotein	FETUA_HUMAN	Positive regulation of ECM constituent secretion; Regulation of ECM assembly; erk 1/2 mitogen-activated protein kinase pathway ANG 2; ANG2 expression of ECM proteins; ANG2 erk1/2 pathway; mek/erk pathway; erk pathway
Angiotensinogen	ANGT_HUMAN	erk pathway
Apolipoprotein A-I	APOA1_HUMAN	erk pathway
Apolipoprotein E	APOE_HUMAN	Positive regulation of ECM constituent secretion
Carboxypeptidase B2	CBPB2_HUMAN	erk pathway
Cathelicidin antimicrobial peptide	CAMP_HUMAN	ras-erk pathway
CD44 antigen	CD44_HUMAN	MMP9 signaling pathway; ras-erk1/2 pathway; mek/erk pathway; erk pathway.
Clusterin	CLUS_HUMAN	Inhibition of ECM disassembly; ECM organization; mapk/erk pathway; MMP9 signaling pathway
Cystatin-C	CYTC_HUMAN	ECM organization
Fibronectin	FINC_HUMAN	ECM organization; Activation of erk pathway; ECM assembly; erk1/2 pathway; Cell–matrix adhesion; erk pathway; Calcium independent cell matrix adhesion; mapk/erk pathway
Fibulin-1	FBLN1_HUMAN	Cell–matrix adhesion
Insulin-like growth factor-binding protein 3	IBP3_HUMAN	erk1/2 pathway
Intercellular adhesion molecule 1	ICAM1_HUMAN	erk1/2 pathway; mek/erk pathway; Cell–matrix adhesion
Kininogen-1	KNG1_HUMAN	Bradykinin in MMP secretion; ECM secretion; mapk/erk pathway
Lumican	LUM_HUMAN	ECM assembly
Pigment epithelium-derived factor	PEDF_HUMAN	MMP secretion; apoptotic signaling pathway; erk1/2 pathway
Transthyretin	TTHY_HUMAN	Apoptotic signaling pathway; erk1/2 pathway
Vitronectin	VTNC_HUMAN	ECM organization; Cell–matrix adhesion
